# The Synthesis and Preclinical Investigation of Lactosamine-Based Radiopharmaceuticals for the Detection of Galectin-3-Expressing Melanoma Cells

**DOI:** 10.3390/pharmaceutics14112504

**Published:** 2022-11-18

**Authors:** Barbara Gyuricza, Ágnes Szűcs, Judit P. Szabó, Viktória Arató, Zita Képes, Dániel Szücs, Dezső Szikra, György Trencsényi, Anikó Fekete

**Affiliations:** 1 Division of Nuclear Medicine and Translational Imaging, Department of Medical Imaging, Faculty of Medicine, University of Debrecen, Nagyerdei krt. 98., H-4032 Debrecen, Hungary; 2 Doctoral School of Chemistry, Faculty of Science and Technology, University of Debrecen, Egyetem tér 1., H-4032 Debrecen, Hungary; 3 Doctoral School of Clinical Medicine, Faculty of Medicine, University of Debrecen, Nagyerdei krt. 98., H-4032 Debrecen, Hungary; 4 Doctoral School of Pharmaceutical Sciences, Faculty of Pharmacy, University of Debrecen, Nagyerdei krt. 98., H-4032 Debrecen, Hungary

**Keywords:** galectin-3, radiolabeling, radiopharmaceutical, PET imaging

## Abstract

Given that galectin-3 (Gal-3) is a β-galactoside-binding lectin promoting tumor growth and metastatis, it could be a valuable target for the treatment of Gal-3-expressing neoplasms. An aromatic group introduced to the C-3′ position of lactosamine increased its affinity for Gal-3. Herein, we aimed at developing a radiopharmaceutical for the detection of Gal-3 positive malignancies. To enhance tumor specificity, a heterodimeric radiotracer capable of binding to both Gal-3 and αvβ3 integrin was also synthetized. Arginine-glycine-asparagine (RGD) peptide is the ligand of angiogenesis- and metastasis-associated αvβ3 integrin. Following the synthesis of the chelator-conjugated (2-naphthyl)methylated lactosamine, the obtained compound was applied as a precursor for radiolabeling and was conjugated to the RGD peptide by click reaction as well. Both synthetized precursors were radiolabeled with ^68^Ga, resulting in high labeling yield (>97). The biological studies were carried out using B16F10 melanoma tumor-bearing C57BL6 mice. High tumor accumulation of both labeled lactosamine derivatives—detected by in vivo PET and ex vivo biodistribution studies—indicated their potential for melanoma detection. However, the heterodimer radiotracer showed high hepatic uptake, while low liver accumulation characterized chelator-conjugated lactosamine, resulting in PET images with excellent contrast. Therefore, this novel carbohydrate-based radiotracer is suitable for the highly selective determination of Gal-3-expressing melanoma cells.

## 1. Introduction

Positron emission tomography (PET) is an effective diagnostic method that uses radioactive agents and plays a vital role in the detection of various cancers even in the early stages of the disease. Since early diagnostic assessment significantly improves the chance of survival, its oncological importance cannot be overemphasized enough. Today, the most commonly applied radiopharmaceutical for the staging of tumor lesions and for the determination of response to anticancer treatment in clinical practice is 2-deoxy-2-[^18^F]fluoro-β-D-glucose ([^18^F]FDG). However, increased [^18^F]FDG uptake is also characteristic for infections and inflammations, which can be misdiagnosed as malignancies [1]. Therefore, there is an urgent need for the development of more tumor-specific radiopharmaceuticals. Radiotracers based on receptor–ligand interaction are capable of binding with high affinity and selectivity to receptors overexpressed in neoplastic cells, which reduces their accumulation in healthy tissues. For example, radiolabeled peptides could be promising candidates regarding tumor lesion detection and the follow-up of anticancer therapies, as well as in targeted radiotherapy settings [2].

In the last decade, galectin-3 as a target came to the forefront of anti-tumor research [3,4,5,6]. The galectin-3 protein is a chimeric beta-galactoside-binding lectin that consists of three distinct parts, an N-terminal domain, a repeating collagen-like sequence, and a C-terminal domain containing the carbohydrate recognition domain (CRD) [3,7], which has affinity for some natural carbohydrate ligands such as lactose, *N*-acetyl-lactosamine (LacNAc) and poly-*N*-acetyl-lactosaminoglycan [8]. Galectin-3 plays a crucial role in various processes of carcinogenesis, such as neoplastic cell adhesion, migration, and invasion, as well as contributing to tumor cell survival and resistance to anticancer therapy [4,6,7,9,10]. According to literature data, several tumor types show positivity for Gal-3 receptor, including colorectal cancer, prostate carcinoma, breast cancer, and melanoma [4,11]. Furthermore, the heightened upregulation of galectin-3 is associated with poor disease prognosis [12,13].

Based on the above-detailed findings, many galectin-3 inhibitors have already been reported for the purpose of preventing and suppressing cancer progression. These inhibitors may possess various chemical bases such as peptides, glycoproteins, and carbohydrates [14,15,16,17,18]. Two different peptides characterized by high galectin-3 affinity—G3-C12 and G3-A9—were identified by Zuo et al. applying combinatorial phage display [19]. Moreover, by investigating the biological activity of these peptides, the inhibition of breast cancer metastasis was observed [19]. Another promising molecule—developed by Safescience—is GBC-590, which is a modified citrus pectin that was reported to reduce colorectal carcinomas in a Phase II trial [20]. The galactomannan derivative GM-CT-01 (DAVANT^®®^) isolated from the seeds of Cyamopsis tetragonolobus serves as a potential drug candidate and has been evaluated in Phase I and II clinical trials for various cancers [21]. In addition to the abovementioned compounds, the preparation of several synthetic Gal-3 ligands, galactose, thiodigalactoside (TDG), and lactosamine (LacNAc) derivatives, has also been published [18,20,22]. Vuong et al. found that 3,4-dichlorophenyl 3-deoxy-3-[4-(3,4,5-trifluorophenyl)-1H-1,2,3-triazol-1-yl]-1-thio-α-D-galactopyranoside (GB1107), a high-affinity ligand of galectin-3, inhibits the growth of lung adenocarcinoma and enhances the effect of immune checkpoint inhibitors [23].

X-ray diffraction measurements were utilized by Seetharaman et al. [8] to evaluate the complex formed by human galectin-3 CRD with lactose and *N*-acetyl-lactosamine (LacNAc). The following interactions were detected. The 4′-OH group of galactose moiety forms a hydrogen bond with amino acids His-158, Asn-160, and Arg-162. Furthermore, the ring oxygen of galactose creates a hydrogen bond with Arg-162 and Glu-184. 6′-OH also interacts with Asn-174 and Glu-184 through hydrogen bonding. In addition, the CH-3′, CH-4′, and CH-5′ of galactose interact with the Trp-181 side chain by van der Waals interactions. While the 3-OH group of the *N*-acetyl-glucose-amine unit forms a hydrogen bond with Glu-184 and Arg-162. *N*-acetyl-lactosamine binds to the CRD of galectin-3 with a 5-fold greater affinity compared to the lactose molecule, since the *N*-acetyl group in position 2 can further interact with Glu-165 and Arg-186 [8].

We previously reported on the preparation of a ^68^Ga-labeled lactosamine-based radiotracer and the investigation of its tumor-targeting and pharmacokinetic characteristics. The in vivo PET and ex vivo biodistribution examinations of this labeled compound were performed in B16-F10 melanoma tumor-bearing mice [24]. However, only moderate tumor radiotracer uptake was observed [24]. The low inhibitory efficiency of this natural ligand could underly our result.

In order to effectively use lactosamine as a galectin-3 inhibitor for therapeutic or diagnostic purposes, its affinity must be increased by chemical modification. Such developments primarily focus on chemical modification of the galactose unit at the C-3′ position, as the 3′-OH group points into the extended groove of the CRD. Accordingly, the extension of the lactosamine at the C-3′ position enables additional interactions with the galectin-3 protein and increases the selectivity of the ligand Gal-3 compared with galectin-1 [25]. Synthetizing several LacNAc derivatives substituted in the C-3′ position and investigating their Gal-3 affinity, Sörme and co-workers [26] found that three compounds bearing an aromatic amide in the C-3′ position showed significantly higher inhibitory activity. Later, they evaluated the human Gal-3 CRD in complex with the ligands containing the 3′-benzamide and 3′-p-methoxy-2,3,5,6-tetrafluorobenzamide groups by X-ray crystallography [27]. Based on these measurements, they established that the benzamide group can interact with the Arg-144 side chain in subunit B of the CRD and causes a conformational change in the side chain. Furthermore, the benzamido ring sits in a nonpolar pocket formed by the side chains of Arg-144, Ala-146 and Asn-160. In addition, a third interaction is in favor of the formation of strong binding, as the guanidino group of Arg-144 can interact with the benzamide group via the cation-Π interaction (also called arginine-arene inter action) [27]. These findings support that the affinity of *N*-acetyl-lactosamine-based galectin-3 inhibitors can be effectively increased by introducing an aromatic substituent at the C-3′ position of the galactose unit.

Despite the fact that many Gal-3 ligands have been prepared, only a few radiolabeled derivatives are known from the available literature. Deutscher et al. [28,29] reported about the synthesis of the radiolabeled analog of G3-C12 peptide, named ^111^In-DOTA-(GSG)-G3-C12 and its preclinical investigation. This radiolabeled peptide—assessed in the following two different xenograft tumor models: MDA-MB-435 human breast carcinoma [28] and PC3-M human prostate carcinoma cells [29]—appeared to be a valuable radiopharmaceutical for the imaging of Gal-3 positive tumors by SPECT. D’Alessandria et al. developed the ^89^Zr-DFO-mAb to Gal-3 radiotracer, which was used to evaluate Gal-3 expression in thyroid carcinoma models by in vivo immunoPET imaging and served specific tumor accumulation [30]. Moreover, radiofluorinated analogs of established Gal-3 inhibitors, namely 1,1′-sulfanediyl-bis-{3-deoxy-3-[4-(3-fluorophenyl)-1H-1,2,3-triazol-1-yl]-β-D-galactopyranoside} (TD139) and GB1107, have been synthetized and applied in PET studies [31]. According to the in vivo distribution studies, the TD139 surrogate showed rapid clearance from the blood, whereas the GB1107 surrogate was characterized by slow elimination; consequently none of the ^18^F-labeled compounds proved to be a suitable PET tracer for the detection of Gal-3 level in pancreatic carcinoma [31]. Hence, to the best of our knowledge, a carbohydrate-based PET radiotracer capable of detecting Gal-3 expression in tumor cells has not yet been described so far.

Therefore, we intended to accomplish the synthesis of a ^68^Ga-labeled lactosamine-based radiopharmaceutical and investigate its Gal-3-targeting properties by in vivo PET imaging and biodistribution studies. In addition, a heterodimeric labeled compound capable of binding to both galectin-3 and α_v_β_3_ integrin was developed to enhance tumor specificity. α_v_β_3_ integrin is a known biomarker of tumor angiogenesis that increases tumor growth and the metastatic potential of tumor cells as well [32]. Peptide analogue containing arginine-glycine-asparagine (RGD) tripeptide sequence binds with high affinity and selectivity to α_V_β_3_ integrin receptor [33]. Consequently, we used a cyclic RGD analog as a vector molecule for the synthesis of the heterodimer radiotracer.

## 2. Materials and Methods

### 2.1. General

The chemicals used for the experiments were obtained from Sigma-Aldrich except for 2,2′,2″-(10-(1-carboxy-4-((4-isothiocyanatobenzyl)amino)-4-oxobutyl)-1,4,7,10-tetraazacyclododecane-1,4,7-triyl) triacetic acid (p-SCN-DOTAGA), which was purchased from ChemaTech (Dijon, France), and cyclic arginine-glycine-asparagine-D-phenylalanine-lysine (cRGDfK), which was from CASLO ApS (Lyngby, Denmark). Kieselgel 60 F254 plates (Merck, Kenilworth, NJ, USA) was used for thin-layer chromatography with UV detection. For radio-TLC, iTLC chromatography paper (Agilent, Santa Clara, CA, USA) was applied and detected by miniGITA TLC scanner (Elysia-raytest, Straubenhardt, Germany). ^1^H (400 MHz) and ^13^C NMR (128 MHz) measurements were recorded on Bruker DRX-400 spectrometer (Bruker, Billerica, MA, USA) using TMS and CDCl_3_ as internal references. LC-MS was carried out a maXis II UHR ESI-QTOF MS Bruker instrument (Bruker, Billerica, MA, USA) and a Waters Acquity UPLC Iclass system (Waters, Milford, CT, USA) were used. ^68^Ga radioisotope was produced by a GE PETtrace cyclotron at Division of Nuclear Medicine, Department of Medical Imaging, University of Debrecen, Hungary. ZR and TK resins were purchased from TrisKem (Bruz, France). CAPINTEC CRC-15PET dose calibrator and a Perkin Elmer Packard Cobra gamma counter (Llantirsant, UK) were applied for determination of radioactivity. Radio-HPLC were performed on Waters 2695 Alliance HPLC system, while semipreparative RP HPLC was carried out in a Waters LC Module 1 HPLC system. Besides UV detection, the radioactivity was measured by ATOMKI CsI scintillation detector. Luna C18 10 µm (250 × 10 mm) column was applied for semipreparative RP HPLC using the following solvent system: A: 0.1% HCOOH; B: 95% acetonitrile. Radio-HPLC was carried out with a Kinetex XB-C18 2.6 μm (50 × 4.60 mm) column, solvent system was A: oxalic acid (0.01 M pH = 3); B: 95% acetonitrile. HPLC-MS grade ACN and MeOH (Fisher Solutions, El Cajon, CA, USA) and deionized water (Milli-Q, 18.2 MΩcm−1, Merck, Kenilworth, NJ, USA) were used for HPLC analysis. The labeled compounds were purified with Oasis HLB 1 cc cartridge (Waters Corporation, Milford, MA, USA).

### 2.2. Chemistry

#### 2.2.1. 3-Azidopropyl-(3-O-(2-naphtyl)methyl-β-D-galactopyranosyl)-(1→4)-(2-*N*-trichloroacetyl-2-deoxy-β-D-glucopyranoside) (**2**)

3-azido-propyl-β-D-galactopyranosyl-(1→4)-(2-*N*-trichloroacetyl-2-deoxy)-β-D-glucopyranoside (1) [24] (118 mg, 0.20 mmol) was dissolved in the mixture of anhydrous toluene (5 mL) and dry MeOH (3 mL). Then dibutyltin-oxide (67 mg, 0.27 mmol) was added to the solution. After stirring over 3 h at 80 °C, the reaction mixture was evaporated. The crude compound was dissolved in dry DMF (5 mL), then to this solution were added 2-(bromomethyl)naphthalene (88 mg, 0.4 mmol) and cesium-fluoride (61 mg, 0.4 mmol). The reaction mixture was stirred overnight at room temperature, then was evaporated. The crude product was purified by column chromatography (Silica gel: 15 g, eluent: CH_2_Cl_2_-MeOH 9:1) to yield **2** (120 mg, 85%) as a colourless syrup. ^1^H NMR (400 MHz, Methanol-d_4_) δ 7.80 (dq, J = 6.5, 2.7 Hz, 4H), 7.51-7.37 (m, 3H), 4.89 (d, J = 12.0 Hz, 1H), 4.79 (d, J = 12,0 Hz, 1H), 4.57 (d, J = 8.1 Hz, 1H), 4.41 (d, J = 7.8 Hz, 1H), 4.02 (d, J = 3,2 Hz 1H), 3.98—3.60 (m, 8H), 3.49—3.44 (m, 1H), 3.42 (dd, J = 9.7, 3.2 Hz 1H), 3.34 (t, J = 6.8 Hz, 2H), 1.84—1.69 (m, 2H). (Supplementary Material Figure S1) ^13^C NMR (Methanol-d_4_): 164.36 (CO), 136.64—125.96 (aromatic C), 104.70 and 101.72 (C-1 and C-1′), 81.79, 81.18, 76.51, 76.01, 72.97, 71.52 and 66.86 (C-2′, C-3, C-3′, C-4, C-4′, C-5 and C-5′), 72.40 (NAPCH_2_), 67.06 and 65.05 (CH_2_), 62.31 and 61.71 (C-6 and C-6′), 58.28 (C-2), 29.73 (CH_2_). (Supplementary Material Figure S2) HRMS ESI calcd for: C_28_H_35_Cl_3_N_4_O_11_, 731.1260 [M+Na]^+^. Found: 731.1261 [M+Na]^+^ (Supplementary Material Figure S6).

#### 2.2.2. 3-Azidopropyl-(3-O-(2-naphtyl)methyl-β-D-galactopyranosyl)-(1→4)-(2-amino-2-deoxy-β-D-glucopyranoside) (**3**)

To a solution of compound **2** (83 mg, 0.117 mmol) in MeOH (2 mL), was added 1 M NaOH (2 mL). The reaction mixture was stirred over 2 days. After stirring the reaction mixture was neutralized with 1 M HCl (2 mL) and evaporated. The crude product was purified by column chromatography (Silica gel: 6 g, eluent: CH_2_Cl_2_-MeOH 8:2) to yield **3** (52 mg, 79%) as a colourless syrup. ^1^H NMR (400 MHz, deuterium-oxide): δ 7.99—7.79 (m, 4H), 7.66—7.42 (m, 3H), 5.00—4.65 (m, 2H), 4.43-4.31 (m, 2H), 4.06 (d, J = 3.2 Hz, 1H), 4.00—3.23 (m, 15H), 2.68 (q, J = 8.6, 7.7 Hz, 1 H), 1.91—1.84 (m, 2H) (Supplementary Material Figure S3). ^13^C NMR (deuterium-oxide): 136.32—127.15 (aromatic C), 104.29 and 103.74 (C-1 and C-1′), 81.34, 80.01, 76.41, 76.05, 75.17, 71.41 and 66.62 (C-2′, C-3, C-3′, C-4, C-4′, C-5, and C-5′), 72.48 (NAPCH_2_), 68.13 (CH_2_), 62.14 and 61.33 (C-6 and C-6′), 57.29 (C-2), 29.46 (CH_2_) (Supplementary Material Figure S4). HRMS ESI calcd for: C_26_H_36_N_4_O_10_, 565.2504 [M+H]^+^. Found: 565.2499 [M+H]^+^ (Supplementary Material Figure S7).

#### 2.2.3. DOTAGA-LacN(NAP) (**4**)

Compound **3** (9.5 mg, 0.017 mmol) was dissolved in the mixture of dry DMSO (600 µL) and 0.1 M Na_2_CO_3_ buffer (pH 9.55, 900 µL). Then to this solution was added p-SCN-Bn-DOTAGA chelating agent (11 mg, 0.017 mmol) in dry DMSO (200 µL). After stirring for two days, water was added to the reaction mixture and concentrated by lyophilization. The purification of the residue was performed on semipreparative RP-HPLC using Luna C18 10 µm (250 × 10 mm) column. The solvent system was A: 0.1% HCOOH and B: 95% acetonitrile. The analysis method was the following: a 32-min gradient from 100% A to 100% B and the flow rate was 4 mL/min. The t_R_ was 17.21 min and the obtained fraction was concentrated by lyophilisation to yield compound 4 (10 mg, 50%). ^1^H NMR (400 MHz, Methanol-d_4_): δ 7.90 (ddd, J = 13.6, 6.3, 2.7 Hz, 5H), 7.61 (dd, J = 8.5, 1.7 Hz, 1H), 7.57—7.45 (m, 2H), 7.38—7.24 (m, 8H), 4.98—4.88 (m, 1H), 4.57—4.24 (m, 1H), 4.09 (d, J = 3.2 Hz, 1H), 4.02—2.86 (m, 33H), 2.59 (dd, J = 20.5, 13.0 Hz, 2H), 2.05—1.95 (m, 2H), 1.87 (d, J = 8.3 Hz 2H) (Supplementary Material Figure S5). HRMS ESI calcd for: C_53_H_74_N_10_O_19_S_1_, 1187.4930 [M+H]^+^ and 594.2504 [M+H]^2+^. Found: 1187.4861 [M+H]^+^, 594.2491 [M+H]^2+^ (Supplementary Material Figure S8).

#### 2.2.4. DBCO-PEG_4_-cRGDfK (**7**)

Compound **5** (4.65 mg, 0.0077 mmol) was dissolved in dimethylsulfoxide (200 µL). Then, *N*,*N*-diisopropylethylamine (6.8 µL, 0.0385 mmol) and a solution of compound **6** (5 mg, 0.0077 mmol) in dimethyl sulfoxyde (200 µL) was added. The reaction mixture was stirred overnight at room temperature. Then water was added to the reaction mixture and concentrated by lyophilization. The purification of the residue was carried out with semipreparative RP-HPLC using the analysis method described for compound **4**. The t_R_ was 17.85 min and the obtained fraction was concentrated by lyophilisation to yield compound **7** (3.9 mg, 44.5%) as a white powder. HRMS ESI calcd for: C_57_H_74_N_11_O_14_, 569.7826 [M+H]^2+^. Found: 569.7839 [M+H]^2+^ (Supplementary Material Figure S9).

#### 2.2.5. DOTAGA-LacN(NAP)-cRGDfK (**8**)

Compound **7** (2 mg, 0.0017 mmol) was dissolved in dimethyl sulfoxide (150 µL). Subsequently, compound **4** (2.5 mg, 0.002 mmol) was also dissolved in dimethyl sulfoxide (150 µL) and added to the solution of compound **7.** The reaction mixture was stirred over night at room temperature. After that, water was added to the reaction mixture and concentrated by lyophilization. The purification of the residue was carried out with semipreparative RP-HPLC using the analysis method described for compound **4**. The t_R_ was 18 min and the obtained fraction was concentrated by lyophilization to yield compound **8** (1 mg, 24.5%) as a white powder. HRMS ESI calcd for: C_110_H_148_N_21_O_33_S_1_, 776.0204 [M+H]^3+^. Found: 776.0251 [M+H]^3+^ (Supplementary Material Figure S10).

#### 2.2.6. DOTAGA-cRGDfK (**9**)

The cRGDfK peptide 5 (10 mg, 0.0165 mmol) was dissolved in 0.1 M Na_2_CO_3_ buffer (pH 9.55, 900 µL. Then the reaction mixture was added p-NCS-Bn-DOTAGA chelating agent (12 mg, 0.0165 mmol) in DMSO (100 µL). After stirring overnight at room temperature, water was added to the reaction mixture and concentrated by lyophilization. The purification of the residue was carried out with semipreparative RP-HPLC using the analysis method described for compound **4**. The t_R_ was 13.95 min, and the obtained fraction was concentrated by lyophilization to afford 2 mg of DOTAGA-cRGDfK (10%). HR-MS (ESI, positive) m/z observed: 613.7855 [M+H]^2+^, 409.5277 [M+H]^3+^; calculated: 613.7853 [M+H]^2+^ and 409.5262 [M+H]^3+^ (see Supplementary Material Figure S11).

### 2.3. Radiochemistry

#### 2.3.1. ^68^Ga labeling of DOTAGA-LacN(NAP), DOTAGA-LacN(NAP)-cRGDfK, and DOTAGA-cRGDfK

^68^Ga isotope was produced in a GE PETtrace cyclotron by ^68^Zn (p, n) ^68^Ga nuclear reaction using 12 MeV proton bombardment of pressed zinc disc (20 mg). The beam current (50 μA) was kept constant, and the irradiation time was 10 min (~3 GBq). The irradiated zinc target was dissolved in 5 M HCl (5 mL), and the obtained solution was loaded on ZR resin (0.3 mL). The ZR resin was washed with 5 M HCl (5 mL). The ^68^Ga isotope was eluted with 2 M HCl (5 mL) to TK200 resin. This resin was washed with 2 M HCl (5 mL), and fractionally eluted with 0.05 M HCl. The radiochemical synthesis was used the purified and concentrated [^68^Ga]GaCl_3_ solution (400 μL, 130–200 MBq). This solution was mixed with 3 M NH_4_OAc buffer (400 μL, pH 4)), and then DOTAGA-LacN(NAP) (4), DOTAGA-LacN(NAP)-cRGDfK (8), and DOTAGA-cRGDfK (9) (1mg/mL, 20 μL) was added to, respectively. This mixtures were incubated at 95 °C for 15 min. The labeled complexes were purified by Oasis HLB 1 cc cartridge. The radiolabeled ligands were washed with water and eluted with EtOH. The radiochemical purity was investigated by radio-HPLC using Kinetex XB-C18 2.6 μm column and the following method: solvent A: 0.1% oxalic acid, solvent B: 95% acetonitrile, gradient: 0 min: 100% A, 1 min: 100% A, 10 min: 100% B, 11 min: 100% B, 12 min: 100% A.

#### 2.3.2. Determination of log *P* Value of [^68^Ga]Ga-DOTAGA-LacN(NAP), [^68^Ga]Ga-DOTAGA-LacN(NAP)-cRGDfK, and [^68^Ga]Ga-DOTAGA-cRGDfK

The purified [^68^Ga]Ga-DOTAGA-LacN(NAP), [^68^Ga]Ga-DOTAGA-LacN(NAP)-cRGDfK, and [^68^Ga]Ga-DOTAGA-cRGDfK (50 μL, 2–3 MBq) was diluted with 450 μL water, and then 500 μL n-octanol was added to, respectively. The mixtures were shaken vigorously for 5 min and centrifugated (9000 rpm) for 5 min. The radioactivity of the n-octanol phases (20 μL) and aqueous phases (20 μL) was determined by gamma counter.

#### 2.3.3. Determination of In Vitro Stability of [^68^Ga]Ga-DOTAGA-LacN(NAP), [^68^Ga]Ga-DOTAGA-LacN(NAP)-cRGDfK, and [^68^Ga]Ga-DOTAGA-cRGDfK

[^68^Ga]Ga-DOTAGA-LacN(NAP), [^68^Ga]Ga-DOTAGA-LacN(NAP)-cRGDfK, and [^68^Ga]Ga-DOTAGA-cRGDfK (50 μL, 4–5 MBq) were mixed with 50 μL of human serum, oxalic acid (0.01 M), and Na_2_EDTA (0.01 M), respectively. The mixtures were incubated at RT for 0 min, 60, and 120 min and then analyzed with radio-HPLC described above and radio-TLC using iTLC paper and 0.5 M citrate buffer (pH 5.5) as an mobile phase (Supplementary Material Figures S12–S18).

### 2.4. Biology

#### 2.4.1. Cell Culturing

B16-F10 mouse melanoma cells (ATCC, VA, USA) were cultured in DMEM (Gibco™, Thermo Fisher Scientific, Waltham, MA, USA) cell culture medium supplemented with 10% (*v*/*v*) heat-inactivated FBS (fetal bovine serum, Thermo Fisher Scientific, Waltham, MA, USA), 1% (*v*/*v*) antimicotic and antibiotic solution, and 1% (*v*/*v*) MEM non-essential amino acid and vitamin solution (Thermo Fisher Scientific, Waltham, MA, USA), respectively. Cells were cultured under standard culturing conditions (37 °C, 5% CO_2_, and 95% humidity) in a cell culture incubator (CCL-170B-8, ESCO Scientific, Singapore) using T75 flasks (Sarstedt Ltd., Budapest, Hungary). For subcutaneous tumor inoculation, cells were used after five passages, and the trypan blue exclusion assay was used to determine the cell viability.

#### 2.4.2. Animal Housing

Twelve-week-old male C57BL/6 mice (n = 30) were kept in individually ventilated cages (IVC) (Techniplast, Akronom Ltd. Budapest, Hungary) at regulated temperature (26 ± 2 °C) and controlled humidity (55 ± 10%). Artificial lighting was assured in mechanically moderated 12-h circadian cycles. Tap water and semi-synthetic rodent chow (SDS VRF, Animalab Ltd., Budapest, Hungary) were administered ad libitum for the enrolled experimental small animals to maintain their physiological nutrient requirements. All applicable paragraphs of the Hungarian Laws and directions and the regulations of the European Union were taken into account regarding both the maintenance and the treatment of the mice. Experimental animals received human care and authorized by the Ethical Committee for Animal Research, University of Debrecen, Hungary (ethical licence number: 16/2020/DEMÁB).

#### 2.4.3. In Vivo PET Imaging and Image Analysis

In a bid to investigate the tumor-targeting capability of the radiopharmaceuticals concerned, in vivo PET imaging was accomplished 10 ± 1 days post-tumor cell implantation. Both normal control and B16-F10 tumorous mice were iv. administered with 6.8 ± 0.9 MBq [^68^Ga]Ga-DOTAGA-LacN(NAP), [^68^Ga]Ga-DOTAGA-LacN(NAP)-cRGDfK, or [^68^Ga]Ga-DOTAGA-cRGDfK in 120 μL saline through the lateral tail vein. Following a 80-min long incubation time static PET scans of 20 min were acquired from the tumorous area applying miniPET-II preclinical PET scanner (Division of Nuclear Medicine and Translational Imaging, Department of Medical Imaging, Faculty of Medicine, University of Debrecen). Post-reconstruction, BrainCAD image analysis software was employed for manual VOI (volume of interest) placement around the assessed regions. With the aim of providing quantitative data, standardized uptake values (SUVs) were determined utilizing the following formula: SUV = [ROI activity (MBq/mL)]/[injected activity (MBq)/animal weight (g)].

#### 2.4.4. Ex Vivo Biodistribution Studies

After in vivo PET imaging (100 min post injection), control and B16-F10 tumor-bearing mice were euthanized with 5% isoflurane (Forane, AbbVie, Budapest, Hungary; OGYI-T-1414/01). For the assessment of the radiotracer uptake, the radioactivity concentration (%ID/g tissue) of the selected organs was measured with a calibrated gamma counter (Perkin-Elmer Packard Cobra, Waltham, MA, USA) (Supplementary Material Table S1).

#### 2.4.5. Statistical Analysis

MedCalc 18.5 commercial software package (MedCalc 18.5, MedCalc Software, Mariakerke, Belgium) was applied for the statistical analyses. To determine the significance, the following tests were utilized: Student’s two-tailed test, two-way ANOVA, and Mann-Whitney U test. Figures are expressed as mean ± SD. The significance was set to 0.05 (*p* < 0.05) except for otherwise indicated.

## 3. Results and Discussion

### 3.1. Chemistry

Our research team previously established a method for the synthesis of the dual-targeting ^68^Ga-NODAGA-LacN-E[c(RGDfK)]_2_ radiotracer [34]. In the present study, a similar synthetic sequence was utilized for the production of the precursor molecules.

First, a lactosamine derivative that was able to bind to the Gal-3 receptor with higher affinity than lactosamine itself was prepared. According to the previously mentioned X-ray crystallographic studies carried out by Sörme and colleagues [27], we designed the synthesis of a lactosamine containing an aromatic group at the C-3′ position. A (2-naphthyl)methyl group that could be easily formed via stanylene acetal was chosen as an aromatic group. This method was suggested for the synthesis of Gal-3 inhibitors by Sörme et al. [35].

**Figure 1:**
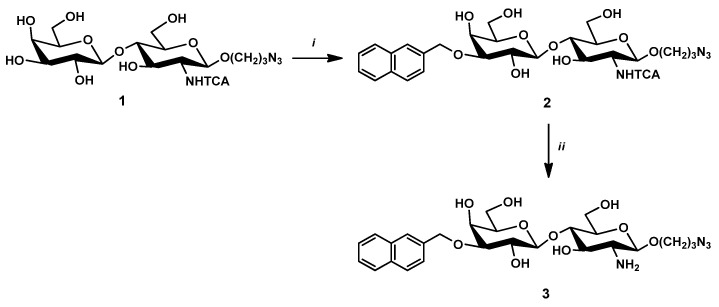
Reagents and conditions: (i) Bu_2_SnO, toluene, reflux, 3 h; NAPBr, CsF, DMF, rt, 24 h 85% for two steps; (ii) NaOH (0.5 M) in MeOH-H_2_O 1:1, rt, 48 h, 79%.

Azido-propyl (β-D-galactopyranosyl)-(1→4)-(2-*N*-trichloroacetyl-2-deoxy-β-D-glucopyranoside (**1**) was the starting material of the synthesis [24]. The 3′-OH group of the galactose unit of lactosamine derivative **1** was regioselectively (2-naphthyl)methylated via stannylene acetal to yield compound **2**. Then, the *N*-trichloroacetyl protecting group was removed by basic hydrolysis, leading to the formation of compound **3** (Figure 1).

**Figure 2:**
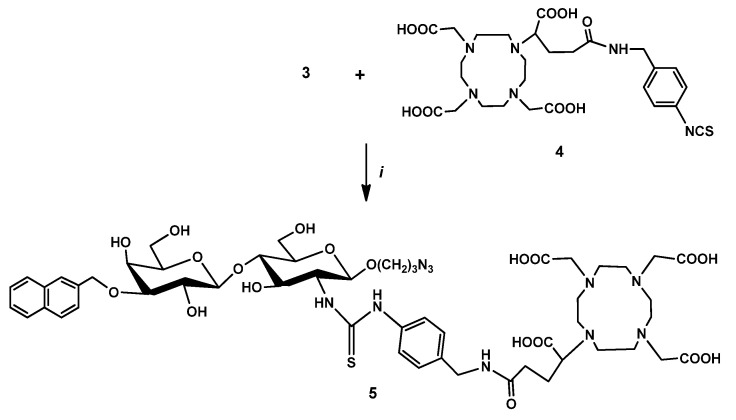
Reagents and conditions (i) DMSO, Na_2_CO_3_ buffer (0.1 M, pH 9.5), rt, 24 h, 28%.

After that, a thiourea bond was formed between the amino group of the (2-naphthyl)methylated lactosamine derivative **3** and the isothiocyanate group of p-SCN-Bn-DOTAGA (**4**) in a mixture of DMSO and 0.1 M sodium carbonate buffer, resulting in compound **5** (Figure 2).

Chelator-conjugated lactosamine derivative **5**—labeled with ^68^Ga isotope—on the one hand was used for biological studies, whereas on the other hand, compound **5** provided one of the building blocks of the desired heterodimer radiopharmaceutical.

**Figure 3:**
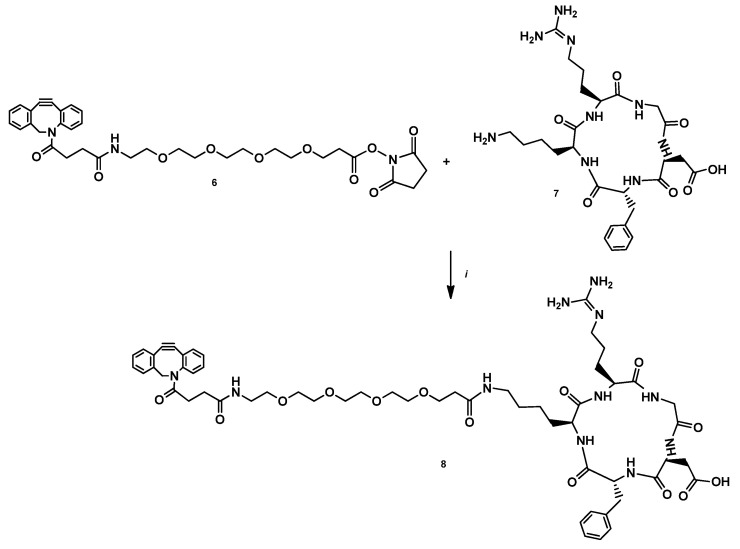
Reagents and conditions: (i) DIPEA, DMSO, rt, 24 h, 44.5%.

Another building block of the heterodimer was a cRGDfK peptide functionalized with a pegylated DBCO unit for the click reaction. Accordingly, the cRGDfK (**7**) peptide was coupled with PEG4-DBCO-NHS (**6**) in a mixture of DMSO and DIPEA to yield compound **8** (Figure 3).

**Figure 4:**
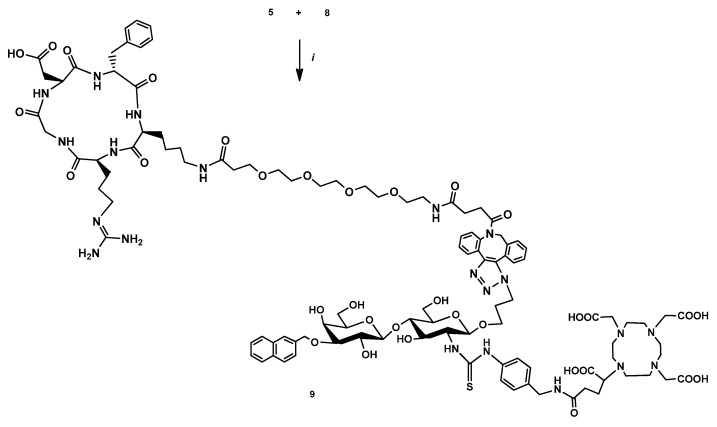
Reagents and conditions: (i) DMSO, rt, 24 h, 24.5%.

Afterwards, the following procedure was applied for the synthesis of heterodimer **9**: the previously prepared chelator-conjugated compound **5** was attached to the compound **8** containing PEG4 and DBCO unit by a copper-free strain-promoted azide–alkyne cycloaddition (SPAAC) (Figure 4).

**Figure 5:**
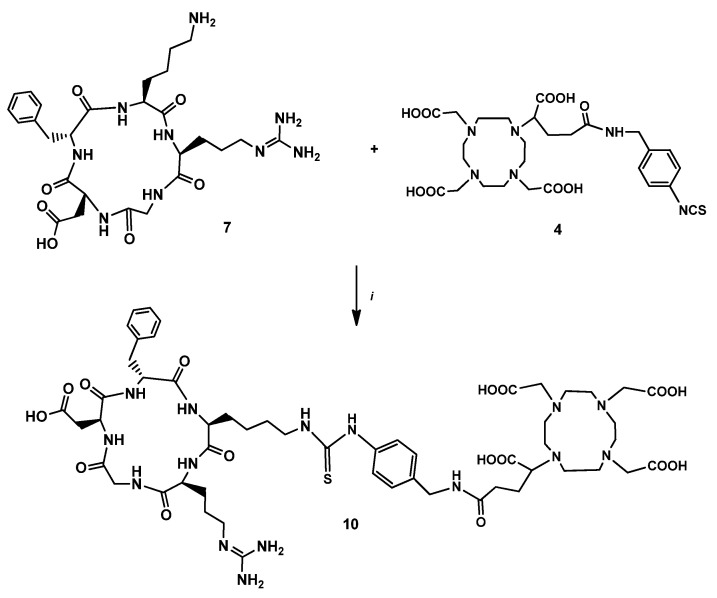
Reagents and conditions: (i) DMSO, Na_2_CO_3_ buffer (0.1 M, pH 9.5), rt, 24 h, 10%.

Finally, a reference compound was prepared for the biological studies. The cRGDfK (**7**) was directly conjugated to the p-SCN-Bn-DOTAGA (**4**) in a mixture of DMSO and 0.1 M sodium carbonate buffer to form DOTAGA-cRGDfK (**10**) (Figure 5).

### 3.2. Radiochemistry

Precursors **5**, **9** and **10** were radiolabeled with ^68^Ga isotope with the application of the same labeling procedure. ^68^Ga nuclide was produced using the ^68^Zn(p, n)^68^Ga nuclear reaction in a cyclotron, and after solid-phase extraction (SPE) it was applied for radiolabeling processes. The obtained [^68^Ga]GaCl_3_ solution (100–200 MBq) was mixed with 3 M NH_4_OAc buffer (pH 4), then a solution of compound **5**, **9** and **10** (concentration: 1 mg/mL) were added to, respectively. These mixtures were incubated at 95 °C for 15 min. In all three cases, high labeling yield was detected (>97%). After SPE purification of the reaction mixtures, the radiochemical purity of the labeled compounds was analyzed by analytical radioHPLC and found to be better than 95% for all radiotraces. In addition, the molar activities were 4.75 ± 0.057 GBq/μmol, 11.63 ± 0.17 GBq/μmol and 5.49 ± 0.19 GBq/μmol for [^68^Ga]Ga-DOTAGA-LacN(NAP), [^68^Ga]Ga-DOTAGA-LacN(NAP)-cRGDfK, and for [^68^Ga]Ga-DOTAGA-cRGDfK, respectively.

Subsequently, the n-octanol/water partition coefficients (logP) of the ^68^Ga-labeled radiotracers were measured. The log *P* values were: −2.63 for [^68^Ga]Ga-DOTAGA-LacN(NAP), −2.27 for [^68^Ga]Ga-DOTAGA-LacN(NAP)-cRGDfK, and −3.03 for [^68^Ga]Ga-DOTAGA-cRGDfK. These results indicated the hydrophilic character of the radiotracers. Furthermore, these log *P* values showed that conjugation with (2-naphthyl)methylated lactosamine **3** and pegylation did not increase but rather decreased the hydrophilicity of the labeled heterodimeric compound.

Furthermore, the labeled complexes were mixed with human serum, 0.01 M oxalic acid and 0.01 M Na_2_EDTA and incubated at room temperature, respectively. Samples from the solutions at 0, 60 and 120 min were analyzed by radio-HPLC. All three radiotracers remained stable under the tested conditions for two hours.

### 3.3. Biology

**Figure 6:**
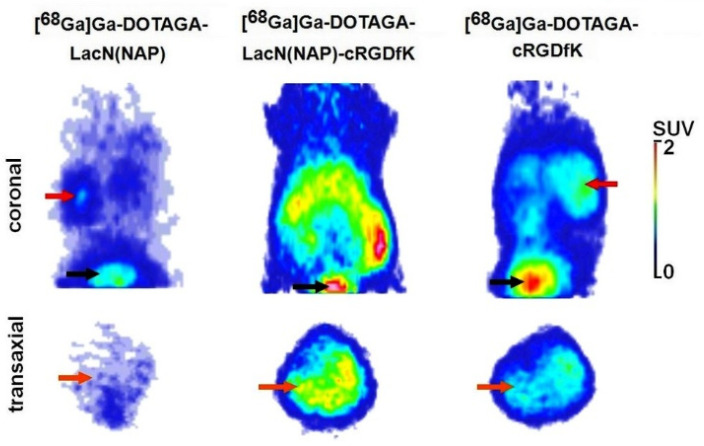
In vivo assessment of [^68^Ga]Ga-DOTAGA-LacN(NAP), [^68^Ga]Ga-DOTAGA-LacN(NAP)-cRGDfK, and [^68^Ga]Ga-DOTAGA-cRGDfK accumulation in healthy control C57BL/6 mice. Representative decay-corrected coronal (**upper row**) and transaxial (**lower row**) PET images were obtained 80 min after intravenous injection of the radiotracers. Red arrows: kidney; black arrows: bladder (urine); orange arrows: liver.

Next, 80 and 100 min post-administration of the radiotracers, in vivo PET examinations and ex vivo tissue distribution studies were conducted to deduce the biodistribution of [^68^Ga]Ga-DOTAGA-LacN(NAP), [^68^Ga]Ga-DOTAGA-LacN(NAP)-cRGDfK, and [^68^Ga]Ga-DOTAGA-cRGDfK in both healthy control and B16-F10 tumorous C57BL/6 mice. Illustrative decay-corrected PET images of healthy mice are presented in Figure 6. Following the qualitative assessment of the PET images, the urinary system (urinary bladder with urine) was clearly visualized (Figure 6 red and black arrows) due to the log *P* values, which confirmed the highly hydrophilic properties of the investigated radiotracers. The high lipophilicity of the radiopharmaceutical causes hepatobiliary excretion and high nonspecific uptake in healthy tissues, which limits its use in imaging and therapy. Therefore, it is important to reduce the lipophilicity of radiolabeled peptides, which increases the tumor-to-background ratio. However, too high hydrophilicity may result in short circulation half-life of the radiopharmaceutical, leading to low accumulation in tumors. However, despite the low log *P* values, as the lower row of Figure 6 demonstrates, increased hepatic radiopharmaceutical accumulation was observed with the RGD-containing radiotracers. Further, comparing the liver tracer uptakes of the three different radioisotopes, the most elevated accumulation was registered in connection with radiotracer [^68^Ga]Ga-DOTAGA-LacN(NAP)-cRGDfK.

**Figure 7:**
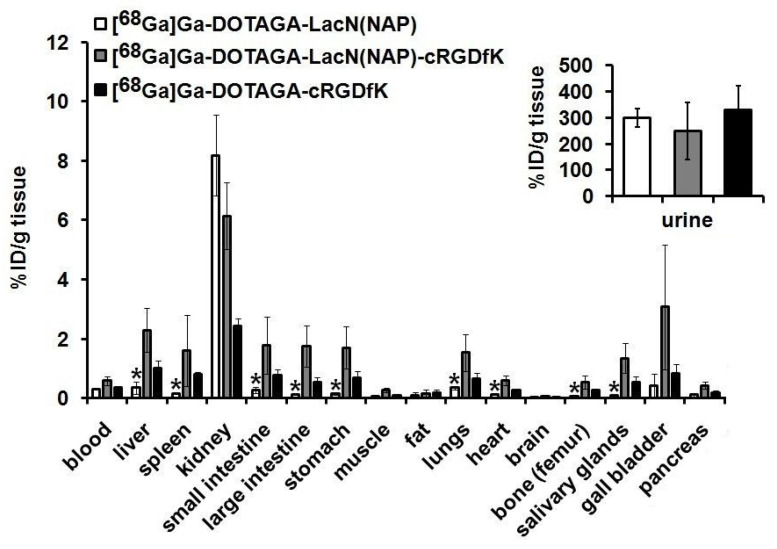
Ex vivo biodistribution of the ^68^Ga-labeled DOTAGA-LacN(NAP), DOTAGA-LacN(NAP)-cRGDfK, and DOTAGA-cRGDfK in C57BL/6 mice (n = 5/radiopharmaceutical) 100 min after intravenous injection of the radiotracers. %ID/g tissue values are presented as mean ± SD. Significance level between the [^68^Ga]Ga-DOTAGA-LacN(NAP) and the two other investigated radiotracers: *p* ≤ 0.01 (*).

The above-detailed in vivo PET results were in line with the ex vivo data (as demonstrated in Figure 7). As part of the ex vivo biodistribution studies—after PET imaging—the sacrifice of the experimental animals occurred followed by the gamma counter-based measurement of the radioactivity of the organs and tissues (Figure 7). In line with the in vivo SUV data, in the case of all three radiotracers, remarkable accumulation was observed in the kidneys (approx. %ID/g: 2–8) and in the urine (approx. %ID/g: 300). Comparing the %ID/g data of the abdominal, thoracic, and other organs, while ^68^Ga-labeled DOTAGA-LacN(NAP) showed (*p* ≤ 0.01) the lowest values, [^68^Ga]Ga-DOTAGA-LacN(NAP)-cRGDfK accumulation was the highest in most of the examined tissues. This elevated accumulation identified in the stomach (%ID/g: 1.70 ± 0.71), liver (%ID/g: 2.30 ± 0.73), gall bladder (%ID/g: 3.07 ± 2.10), and intestines (%ID/g: approx. 1.70 ± 0.70) indicates that there is an elimination route of LacN(NAP)-cRGDfK-targeted radiopharmaceutical through the digestive system (Figure 7).

The high liver uptake and hepatobiliary excretion of the labeled RGD-containing compounds can be explained by the following. According to prior literature data, due to the size and physicochemical properties of the RGD-containing radiotracers, the reticuloendothelial cells of the liver and the spleen, the vascularization and hepatic metabolism can also increase their accumulation in these organs. In addition, the elevated intestinal uptake may be related to the physiological α_v_β_3_ expression of the intestinal smooth muscle cells [36].

However, low galectin-3 expression was found in the healthy kidney and liver by Chen et al. [37] and Hsu et al. [38]. In accordance with these observations, higher liver accumulation was recorded when α_v_β_3_-specific RGD-containing radioactive tracers were used, while this value was significantly lower in the case of the galectin-3-specific ^68^Ga-labeled DOTAGA-LacN(NAP) probe.

**Figure 8:**
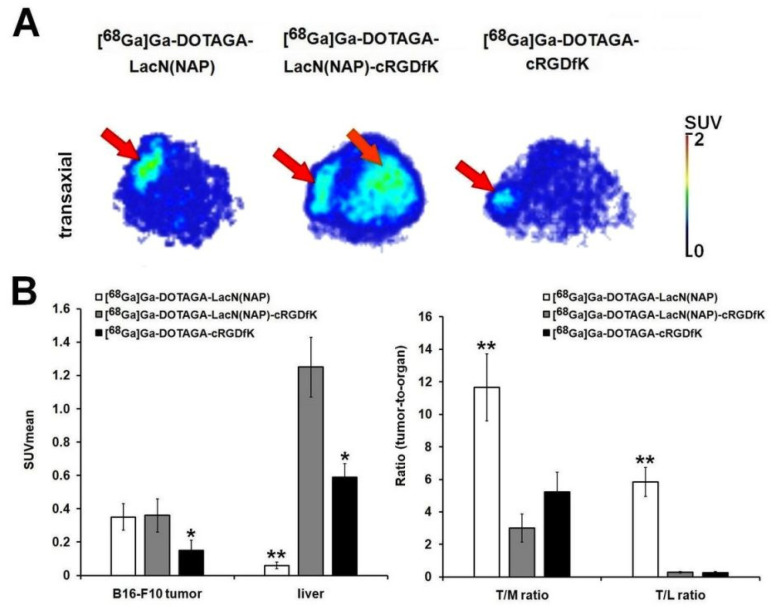
In vivo assessment of ^68^Ga-labeled DOTAGA-LacN(NAP), DOTAGA-LacN(NAP)-cRGDfK, and DOTAGA-cRGDfK accumulation in subcutaneous B16-F10 tumors. Representative decay-corrected transaxial PET images of B16-F10 tumor-bearing mice 80 min after intravenous injection of the radiotracers and 10 ± 1 days after subcutaneous tumor cell inoculation (**A**). Quantitative SUV analysis of radiotracer uptake in B16-F10 tumors (n = 5/radiopharmaceutical) 80 min post injection, and 10 ± 1 days after subcutaneous injection of tumor cells (**B**). Significance levels: *p* ≤ 0.05 (*) and *p* ≤ 0.01 (**). SUV: standardized uptake value; T/M: SUVmean of tumor/SUVmean of muscle. T/L: SUVmean of tumor/SUVmean of liver. SUV values are presented as mean ± SD. Red arrows: B16-F10 tumor; orange arrow: liver.

Prior literature data indicated both α_v_β_3_ integrin [39,40] and galectin-3 receptor [41,42] positivity of B16-F10 melanoma tumors. Initiated by the related findings, in vivo PET and ex vivo biodistribution studies were carried out to assess the tumor targeting properties of ^68^Ga-labeled DOTAGA-LacN(NAP), DOTAGA-LacN(NAP)-cRGDfK, and DOTAGA-cRGDfK in B16-F10 tumor-bearing mice. After the qualitative assessment of the decay-corrected PET images, we found that the subcutaneously growing B16-F10 melanoma tumors could be clearly identified with all the three investigated radiopharmaceuticals (Figure 8A; red arrows). However, given the high hepatic accumulation of the RGD-containing radiopharmaceuticals, the evaluation of the images was difficult. The quantitative SUV analysis of the B16-F10 tumors revealed (*p* ≤ 0.05) the lowest SUVmean (SUVmean: 0.15 ± 0.06) in the case of [^68^Ga]Ga-DOTAGA-cRGDfK. After the injection of the ^68^Ga-labeled DOTAGA-LacN(NAP) and DOTAGA-LacN(NAP)-cRGDfK there no significant difference was depicted between the SUVmean of the B16-F10 tumors (SUVmean: 0.35 ± 0.08 and 0.36 ± 0.10, respectively) (Figure 8B). The SUV mean data of the liver was comparable to the in vivo qualitative observations, since the [^68^Ga]Ga-DOTAGA-LacN(NAP)-cRGDfK presented the highest SUVs (SUVmean: 1.25 ± 0.18). Although, significantly lower accumulation was observed using [^68^Ga]Ga-DOTAGA-LacN(NAP) (SUVmean: 0.06 ± 0.02; *p* ≤ 0.01) and [^68^Ga]Ga-DOTAGA-cRGDfK (SUVmean: 0.59 ± 0.08; *p* ≤ 0.05) radiopharmaceuticals (Figure 8B). Assessing the tumor-to-organ ratios, the [^68^Ga]Ga-DOTAGA-LacN(NAP) probe showed a 2- to 3-fold higher T/M ratio and approximately 21-fold higher T/L ratio than the two other investigated radiotracers (Figure 8B). Consequently, more contrasted PET images of this chelator-conjugated lactosamine derivative could be visualized (Figure 8B).

Similar results were obtained after the ex vivo analysis of the B16-F10 tumors, confirming the outstanding imaging properties of the ^68^Ga-labeled DOTAGA-LacN(NAP) radiopharmaceutical (Table 1).

## 4. Conclusions

We successfully synthesized a ^68^Ga-labeled radiopharmaceutical containing (2-naphtyl)methylated lactosamine for the PET detection of Gal-3 expression in melanoma cells. Furthermore, we accomplished the preparation of a ^68^Ga-labeled heterodimeric compound that can target both galectin-3 and α_v_β_3_ integrin.

Following the radiolabeling processes, the pharmacokinetics and the tumor targeting properties of the synthetized radiotracers were investigated in B16-F10 melanoma tumor-bearing mice applying in vivo PET and ex vivo biodistribution studies. Satisfactory tumor accumulation was experienced in case of both novel (2-naphtyl)methylated lactosamine-containing radiopharmaceuticals. However, despite the glycosylation and pegylation of the RGD peptide, the labeled heterodimer showed high liver uptake and hepatobiliary excretion. Furthermore, despite dual targeting, its tumor uptake was almost identical to that of the Gal-3-specific radiotracer. More enhanced tumor-to-background ratio and higher resolution PET images of the Gal-3 positive tumor cells acquired with the application of the [^68^Ga]Ga-DOTAGA-LacN(NAP) will strengthen the selectivity and imaging properties of this ^68^Ga-labeled carbohydrate derivative compared with those of the heterodimer.

## Figures and Tables

**Table 1:** Ex vivo assessment of the uptake of ^68^Ga-labeled DOTAGA-LacN(NAP), DOTAGA-LacN(NAP)-cRGDfK, and DOTAGA-cRGDfK in experimental subcutaneous B16-F10 tumors 100 min postinjection of the radiopharmaceutical and 10 ± 1 days following tumor cell transplantation. %ID/g tissue values are expressed as mean ± SD. Level of significance (between the T/M and T/L ratios of [^68^Ga]Ga-DOTAGA-LacN(NAP) and the two other radiotracers): *p* ≤ 0.01 (**).T/M: SUVmean of tumor/SUVmean of muscle. T/L: SUVmean of tumor/SUVmean of liver.

Tumor	[^68^Ga]Ga-DOTAGA-LacN(NAP)	[^68^Ga]Ga-DOTAGA-LacN(NAP)-cRGDfK	[^68^Ga]Ga-DOTAGA-cRGDfK
B16-F10 (n = 5)	0.81 ± 0.27	0.80 ± 0.23	0.35 ± 0.12
B16-F10/Muscle ratio	13.97 ± 2.58 **	3.43 ± 1.09	4.54 ± 1.24
B16-F10/Liver ratio	2.66 ± 0.04 **	0.45 ± 0.11	0.35 ± 0.14

## Data Availability

Not applicable.

## Supplementary Materials

The following supporting information can be downloaded at: https://www.mdpi.com/article/10.3390/pharmaceutics14112504/s1, Figure S1: ^1^H NMR spectrum of compound **2**, Figure S2: ^13^C NMR spectrum of compound **2**, Figure S3: ^1^H NMR spectrum of compound **3**, Figure S4: ^13^C NMR spectrum of compound **3**, Figure S5: ^1^H NMR spectrum of compound **4**, Figure S6: Mass spectrum of compound **2**, Figure S7: Mass spectrum of compound **3**, Figure S8: Mass spectrum of compound **4**, Figure S9: Mass spectrum of compound **7**, Figure S10: Mass spectrum of compound **8**, Figure S11: Mass spectrum of compound **9**, Figure S12: Stability test of [^68^Ga]Ga-DOTAGA-LacN(NAP) in 0.01 M Na2EDTA solution, Figure S13: Stability test of [^68^Ga]Ga-DOTAGA-LacN(NAP) in 0.01 M oxalic acid solution, Figure S14: Stability test of [^68^Ga]Ga-DOTAGA-LacN(NAP)-cRGDfK in 0.01 M Na2EDTA solution, Figure S15: Stability test of [^68^Ga]Ga-DOTAGA-LacN(NAP)-cRGDfK in 0.01 M oxalic acid solution, Figure S16: Radio-TLC chromatogram of [^68^Ga]GaCl_3_ solution, Figure S17: Stability test of [^68^Ga]Ga-DOTAGA-LacN(NAP) in human serum after 2 h, Figure S18: Stability test of [^68^Ga]Ga-DOTAGA-LacN(NAP)-cRGDfK) in human serum after 2 h, Table S1: Ex vivo biodistribution data of ^68^Ga-labeled DOTAGA-LacN(NAP), DOTAGA-LacN(NAP)-cRGDfK, and DOTAGA-cRGDfK.
