# Supplementary material for: The Synthesis and Preclinical Investigation of Lactosamine-Based Radiopharmaceuticals for the Detection of Galectin-3-Expressing Melanoma Cells
Source: Pharmaceutics. 2022 Nov 18;14(11):2504. doi: 10.3390/pharmaceutics14112504 (PMC9695418; doi:10.3390/pharmaceutics14112504)
Supplement: Supplementary file 1 [file pharmaceutics-14-02504-s001.zip › pharmaceutics-2001858-supplementary.pdf]

pharmaceutics

MDPI

# Synthesis and preclinical investigation of lactosamine-based radiopharmaceuticals for the detection of galectin-3-expressing melanoma cells

**Barbara Gyuricza, Ágnes Szűcs, Judit P. Szabó, Viktória Arató, Zita Képes, Dániel Szücs, Dezső Szikra, György Trencsényi, Anikó Fekete**

**Part 1: $^{1}$H NMR and $^{13}$C NMR spectra of compound 2, 3 and 4.**

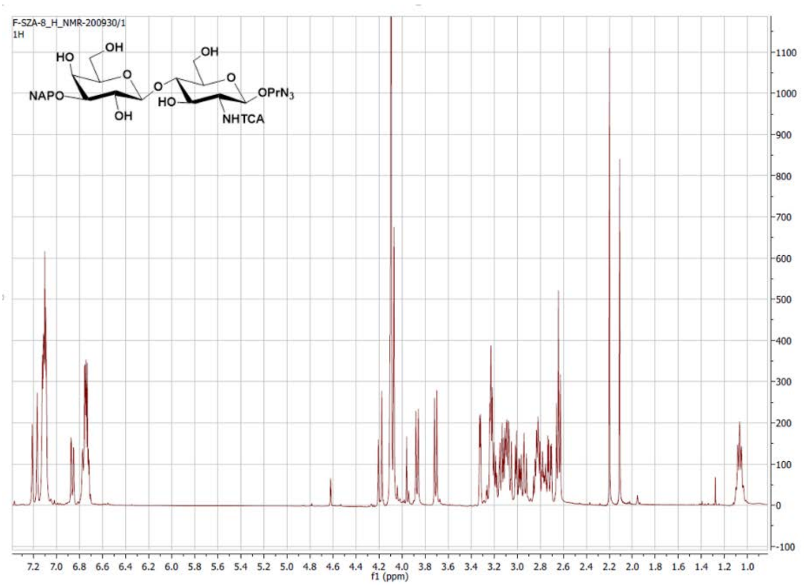


**Figure S1** $^{1}$H NMR spectrum of compound **2**

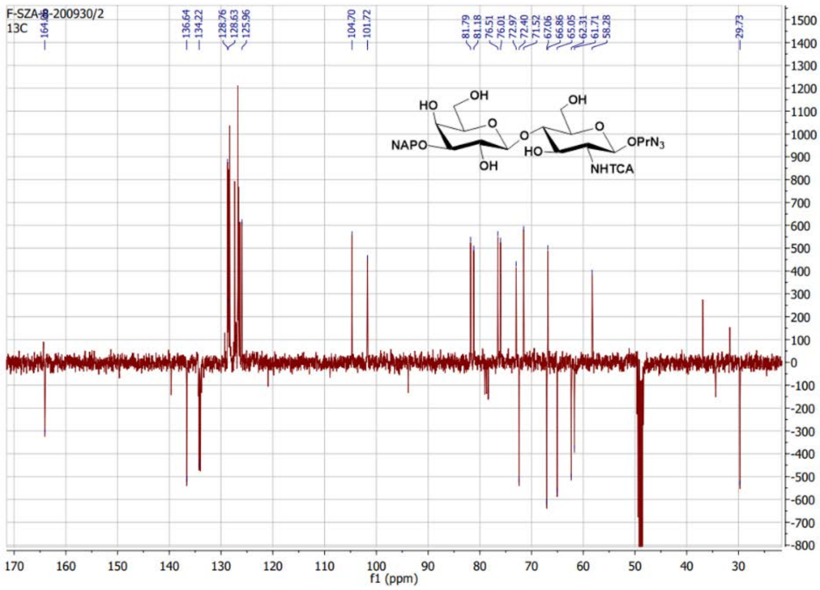


**Figure S2** $^{13}$C NMR spectrum of compound **2**

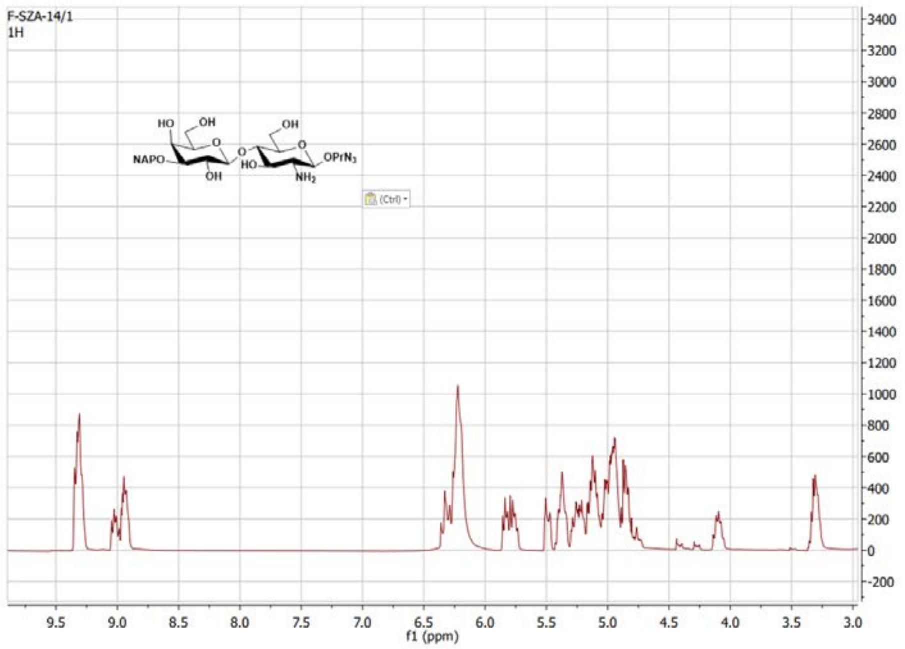


**Figure S3** $^{1}$H NMR spectrum of compound **3**

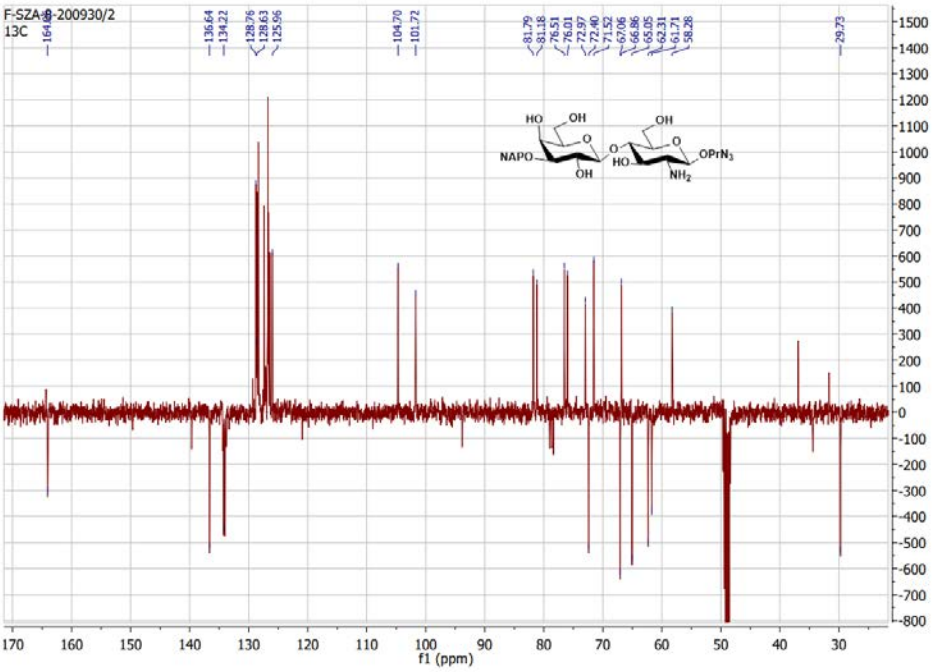


**Figure S4** $^{13}$C NMR spectrum of compound **3**

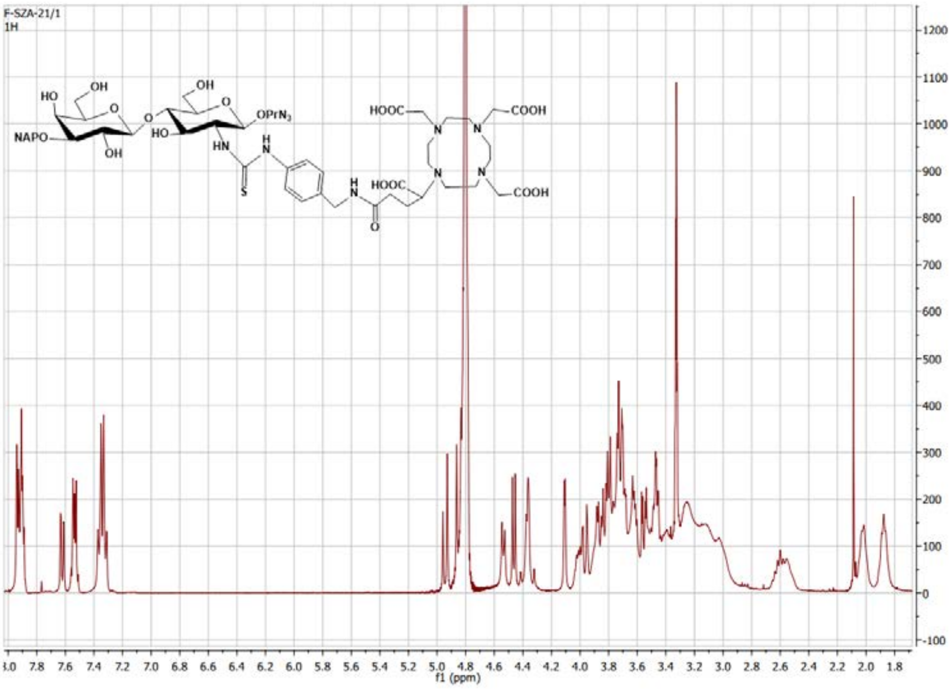


**Figure S5** $^1$H NMR spectrum of compound **4**

**Part 2: Mass spectra of compound 2, 3, 4, 7, 8 and 9**

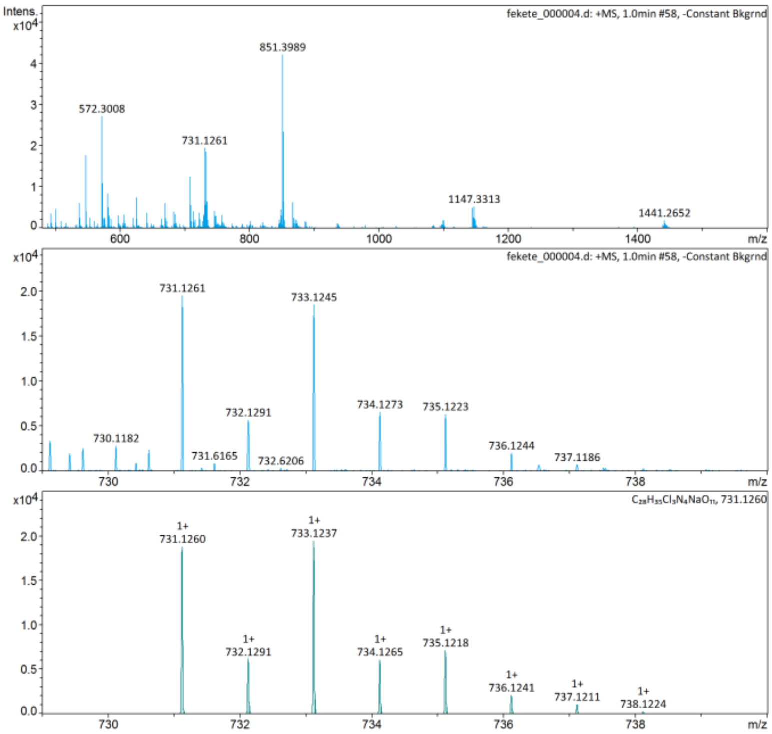


**Figure S6** Mass spectrum of compound **2**

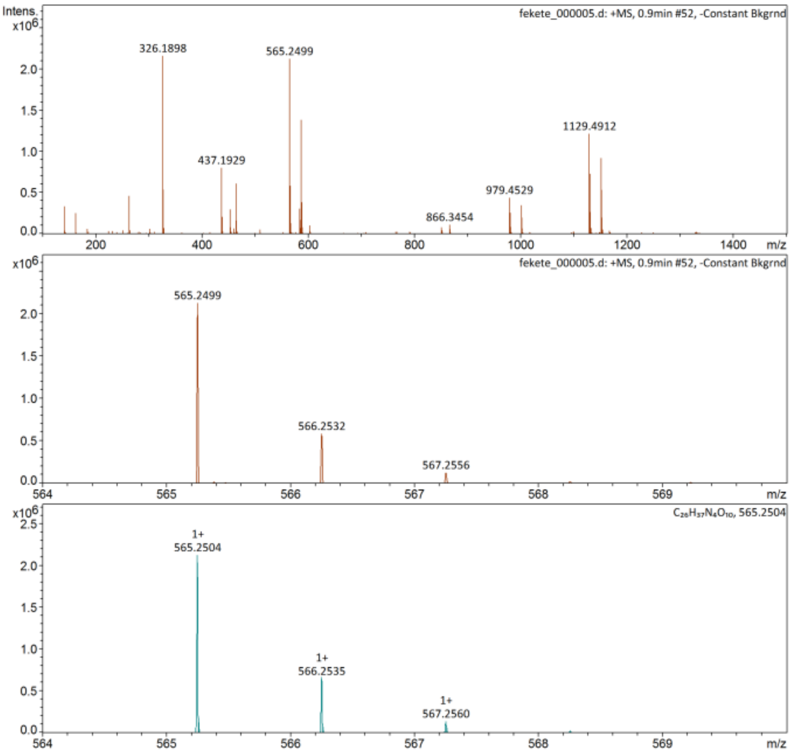


**Figure S7** Mass spectrum of compound **3**

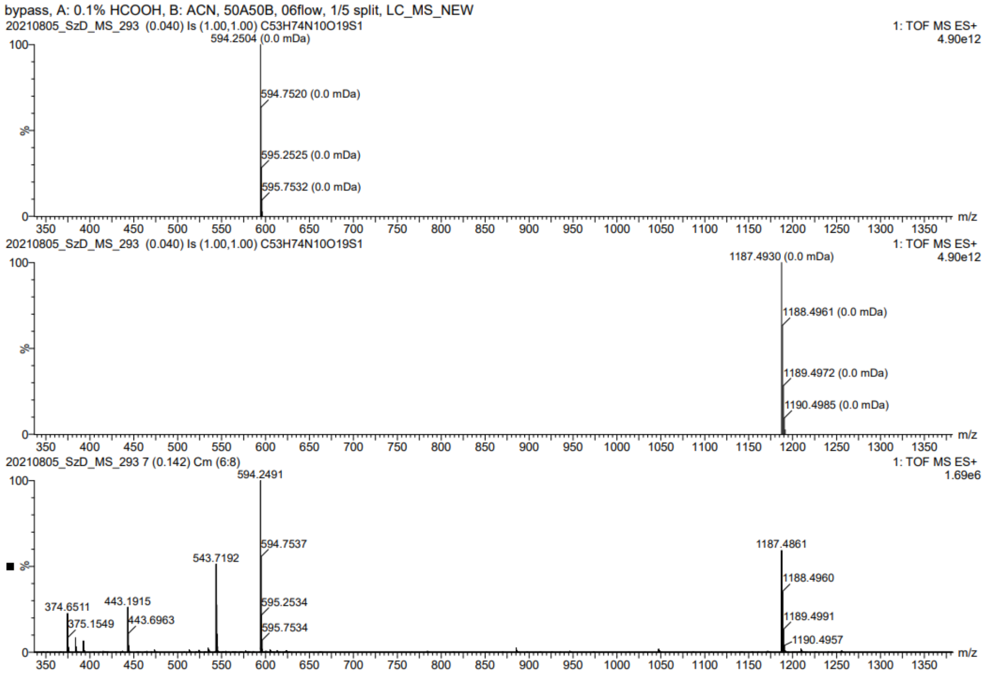


**Figure S8** Mass spectrum of compound **4**

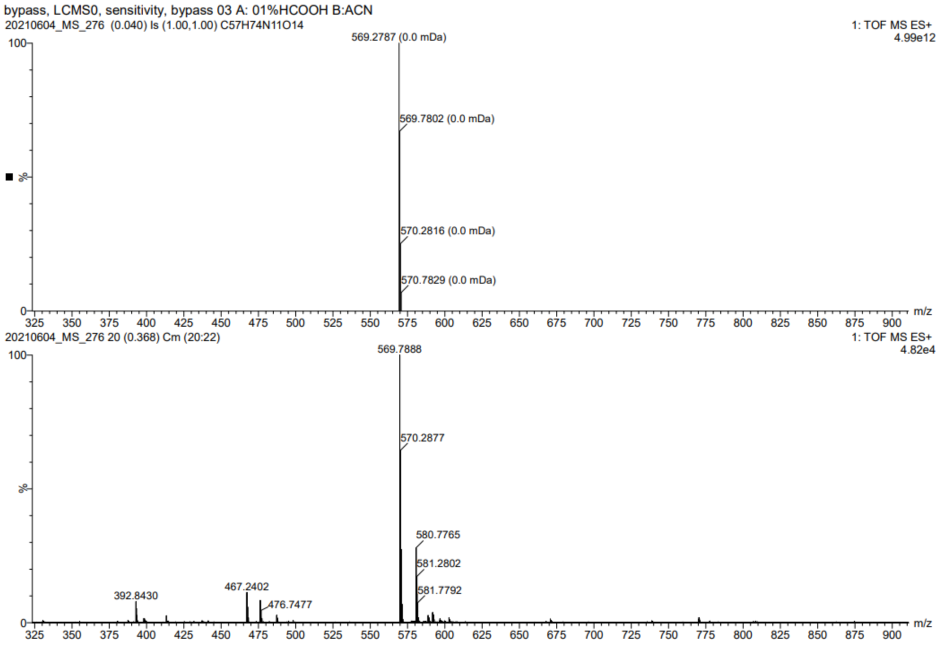


**Figure S9** Mass spectrum of compound **7**

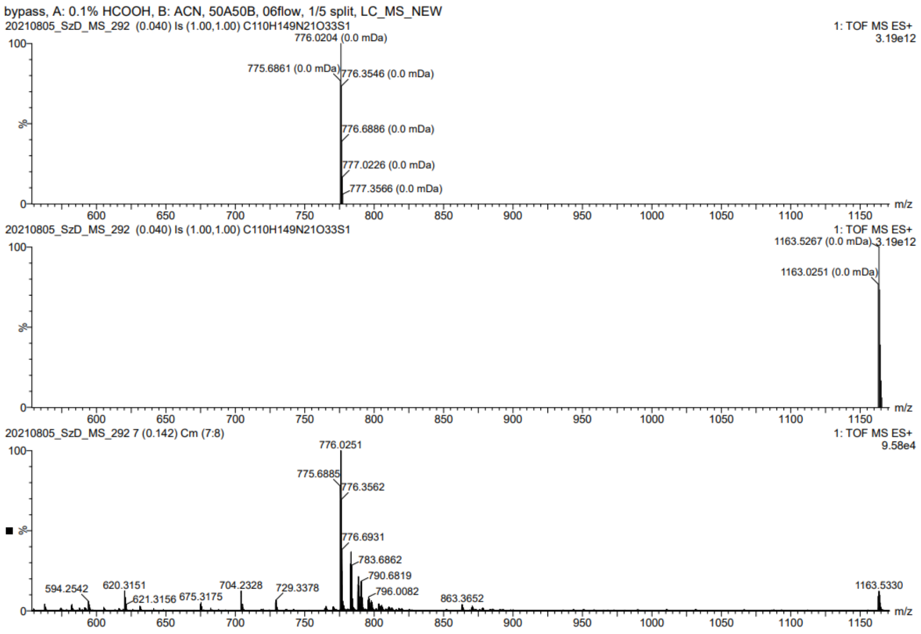


**Figure S10** Mass spectrum of compound **8**

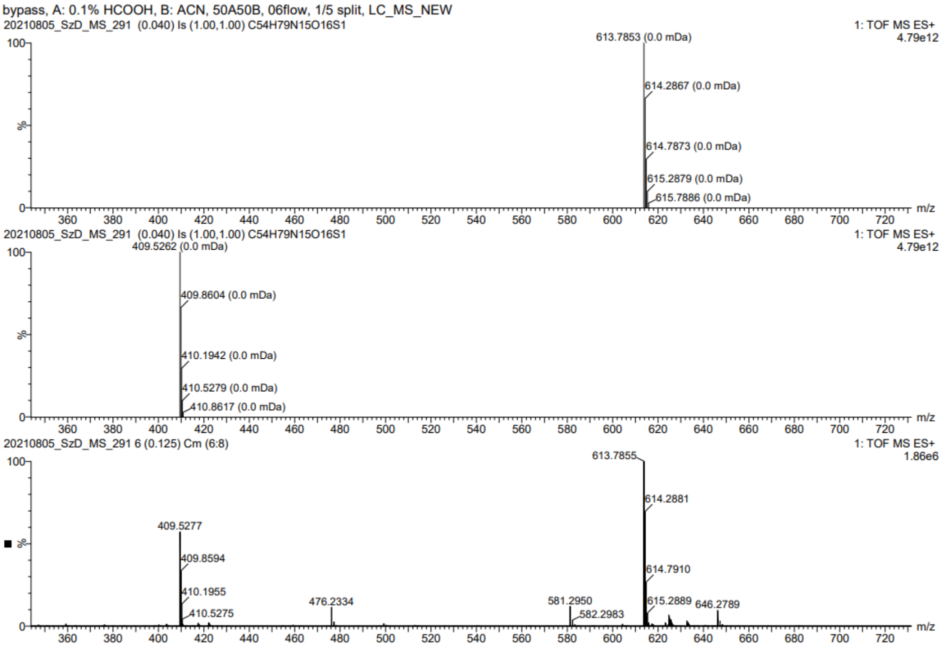


**Figure S11** Mass spectrum of compound **9**

**Part 3: Stability studies of [$^{68}$Ga]Ga-DOTAGA-LacN(NAP) and [$^{68}$Ga]Ga-DOTAGA-LacN(NAP)-cRGDfK**

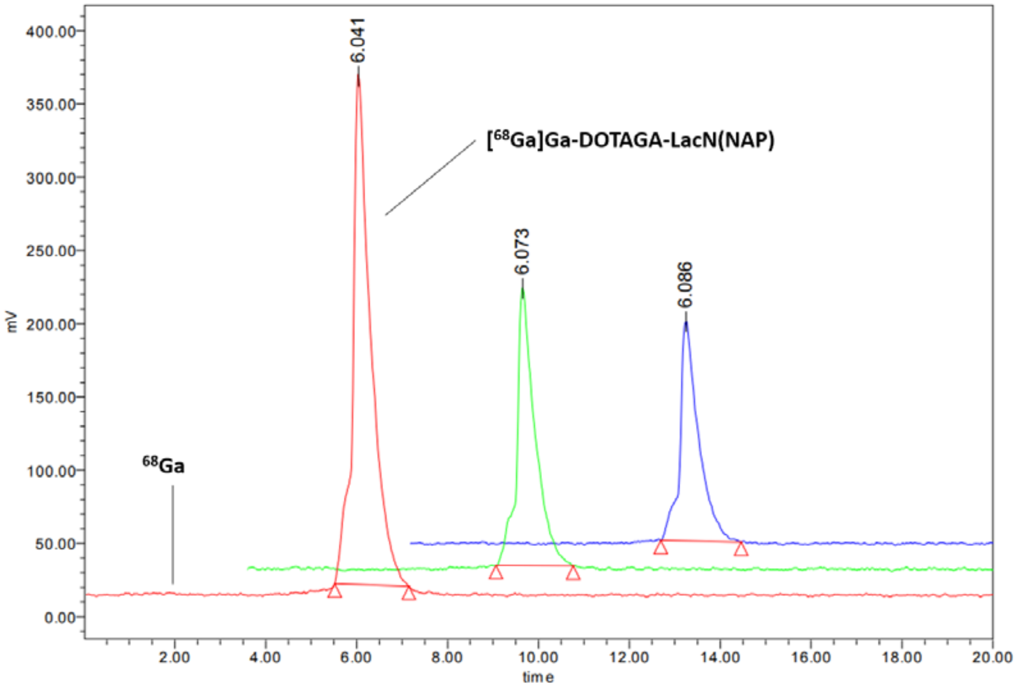


**Figure S12** Stability test of [$^{68}$Ga]Ga-DOTAGA-LacN(NAP) in 0.01 M $Na_2EDTA$ solution.

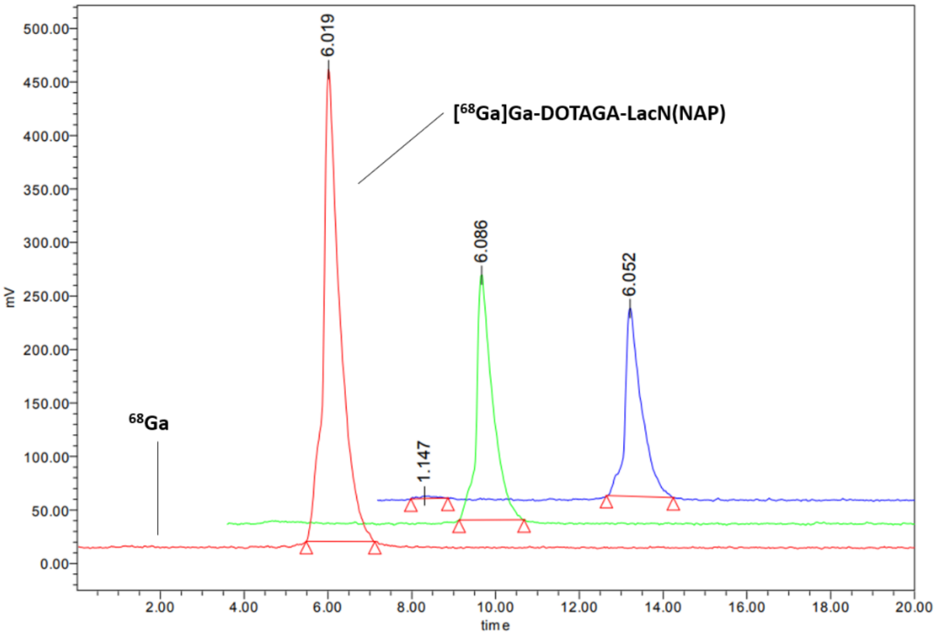


**Figure S13** Stability test of [$^{68}$Ga]Ga-DOTAGA-LacN(NAP) in 0.01 M oxalic acid solution.

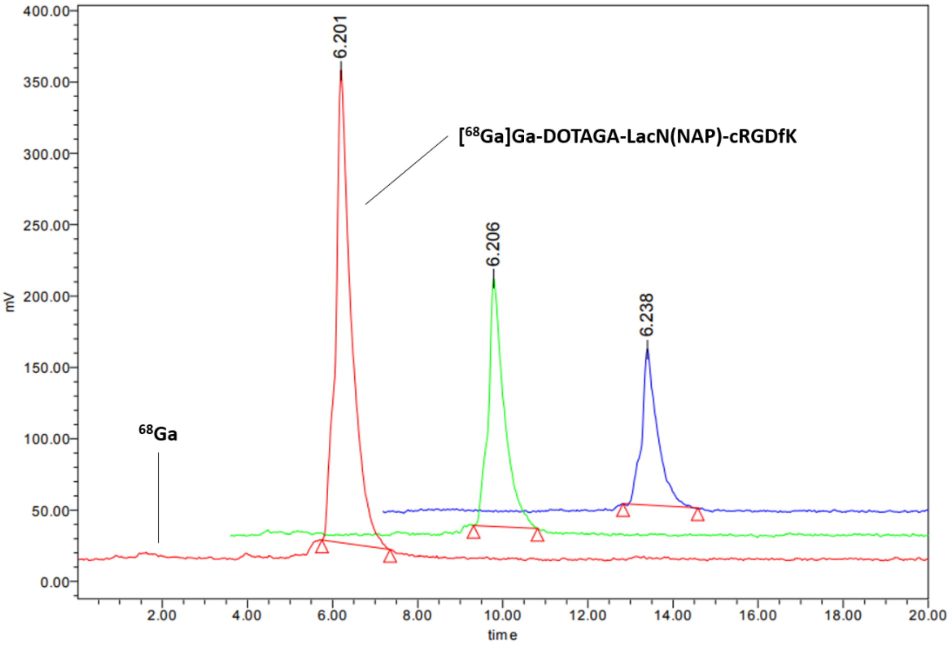


**Figure S14** Stability test of [$^{68}$Ga]Ga-DOTAGA-LacN(NAP)-cRGDfK in 0.01 M $Na_2$EDTA solution.

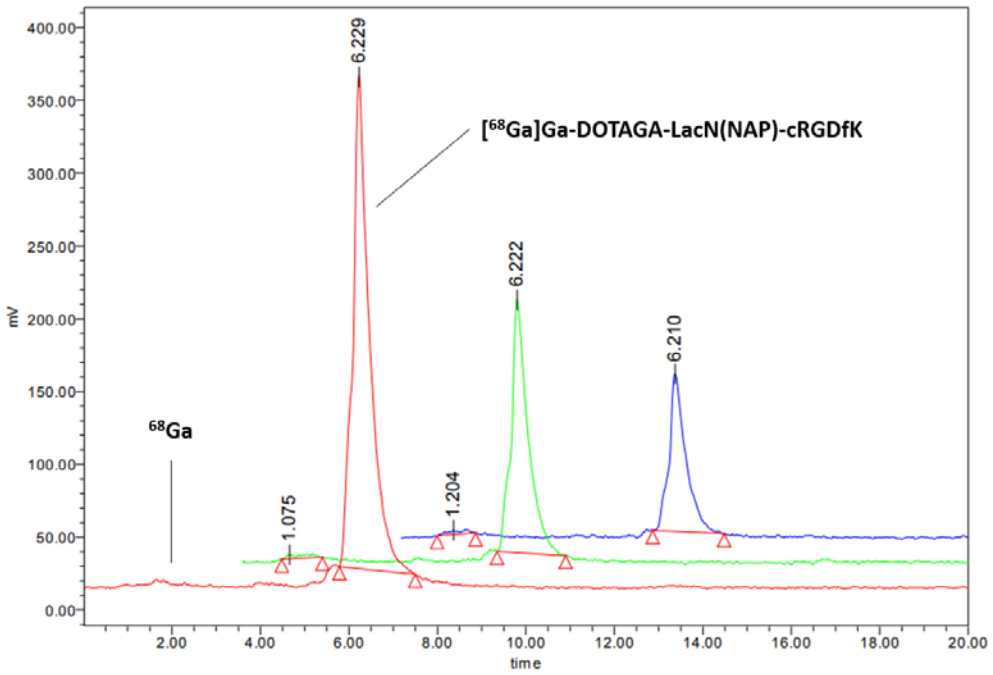


**Figure S15** Stability test of [$^{68}$Ga]Ga-DOTAGA-LacN(NAP)-cRGDfK in 0.01 M oxalic acid solution.

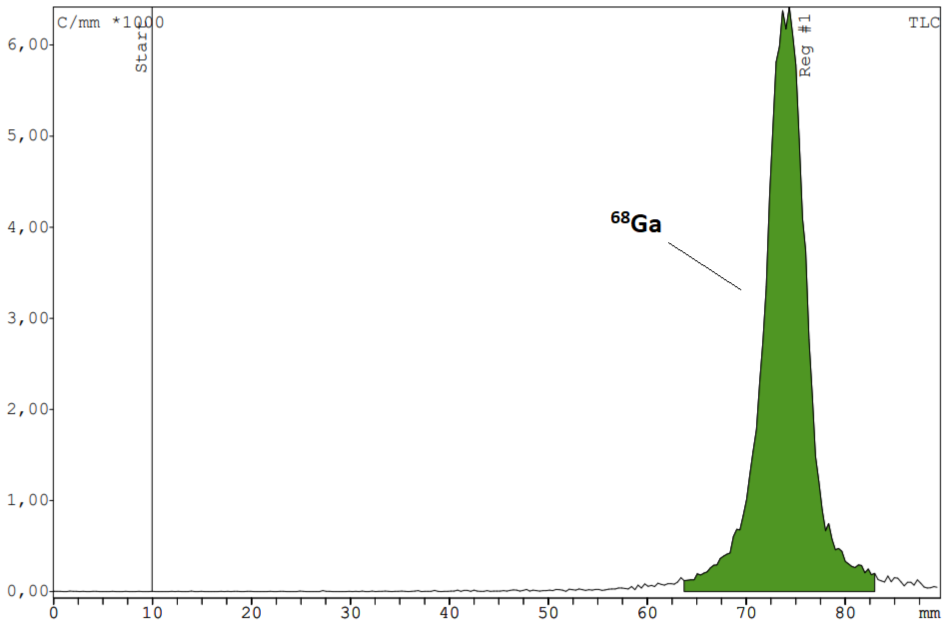


**Figure S16** Radio-TLC chromatogram of [$^{68}$Ga]$GaCl_3$ solution.

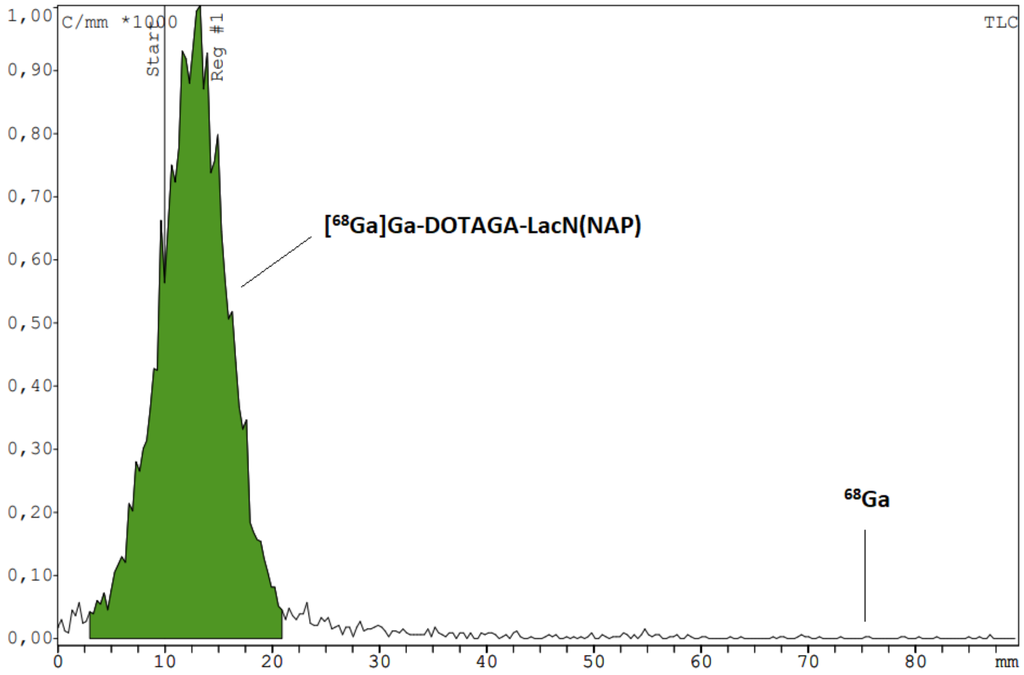


**Figure S17** Stability test of [$^{68}$Ga]Ga-DOTAGA-LacN(NAP) in human serum after 2 hours.

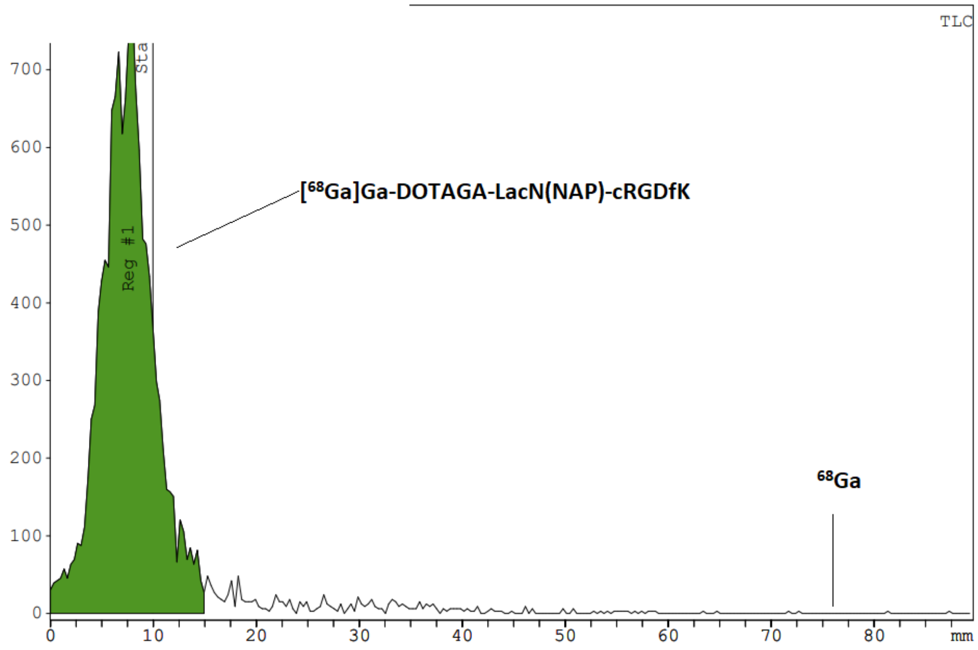


**Figure S18** Stability test of [$^{68}$Ga]Ga-DOTAGA-LacN(NAP)-cRGDfK in human serum after 2 hours.

**TableS1** *Ex vivo* biodistribution of $^{68}$Ga-labeled DOTAGA-LacN(NAP), DOTAGA-LacN(NAP)-cRGDfK, and DOTAGA-cRGDfK in B16-F10 tumor-bearing mice (n=5/radiopharmaceutical) 100 min after intravenous injection of the radiotracers. %ID/g tissue values are presented as mean±SD.

| | **[$^{68}$Ga]Ga-DOTAGA-LacN(NAP)** | **[$^{68}$Ga]Ga-DOTAGA-LacN(NAP)-cRGDfK** | **[$^{68}$Ga]Ga-DOTAGA-cRGDfK** |
|---|---|---|---|
| blood | 0.29±0.01 | 0.58±0.14 | 0.37±0.05 |
| liver | 0.33±0.19 | 2.19±0.73 | 1.00±0.25 |
| spleen | 0.17±0.02 | 1.60±1.17 | 0.80±0.09 |
| kidney | 8.19±1.36 | 6.14±1.14 | 2.43±0.26 |
| small intestine | 0.27±.0.9 | 1.77±0.96 | 0.78±0.20 |
| large intestine | 0.13±0.01 | 1.74±0.70 | 0.55±0.16 |
| stomach | 0.14±0.04 | 1.70±0.72 | 0.69±0.21 |
| muscle | 0.05±0.01 | 0.27±0.07 | 0.10±0.01 |
| fat | 0.11±0.08 | 0.17±0.13 | 0.19±0.08 |
| lungs | 0.35±0.06 | 1.54±0.62 | 0.66±0.18 |
| heart | 0.12±0.02 | 0.61±0.17 | 0.26±0.06 |
| brain | 0.02±0.01 | 0.06±0.01 | 0.03±0.01 |
| bone (femur) | 0.07±0.02 | 0.54±0.21 | 0.26±0.04 |
| salivary glands | 0.11±0.02 | 1.34±0.50 | 0.54±0.18 |
| gall bladder | 0.43±0.38 | 3.07±2.10 | 0.84±0.29 |
| pancreas | 0.11±0.06 | 0.43±0.12 | 0.18±0.06 |
